# Role of dual specificity tyrosine-phosphorylation-regulated kinase 1B (Dyrk1B) in S-phase entry of HPV E7 expressing cells from quiescence

**DOI:** 10.18632/oncotarget.5222

**Published:** 2015-08-19

**Authors:** Na Zhou, Shoudao Yuan, Rongchun Wang, Weifang Zhang, Jason J. Chen

**Affiliations:** ^1^ Cancer Research Center, Shandong University School of Medicine, Jinan, Shandong, China; ^2^ Biology Institute of Shandong Academy of Sciences, Jinan, Shandong, China; ^3^ Institute of Pathogenic Biology, Shandong University School of Medicine, Jinan, Shandong, China

**Keywords:** HPV, E7, quiescence, S-phase, Dyrk1B, p27

## Abstract

The high-risk human papillomavirus (HPV) is the causative agent for cervical cancer. The HPV E7 oncogene promotes S-phase entry from quiescent state in the presence of elevated cell cycle inhibitor p27Kip1, a function that may contribute to carcinogenesis. However, the mechanism by which HPV E7 induces quiescent cells to entry into S-phase is not fully understood. Interestingly, we found that Dyrk1B, a dual-specificity kinase and negative regulator of cell proliferation in quiescent cells, was upregulated in E7 expressing cells. Surprisingly and in contrast to what was previously reported, Dyrk1B played a positive role in S-phase entry of quiescent HPV E7 expressing cells. Mechanistically, Dyrk1B contributed to p27 phosphorylation (at serine 10 and threonine 198), which was important for the proliferation of HPV E7 expressing cells. Moreover, Dyrk1B up-regulated HPV E7. Taken together, our studies uncovered a novel function of Dyrk1B in high-risk HPV E7-mediated cell proliferation. Dyrk1B may serve as a target for therapy in HPV-associated cancers.

## INTRODUCTION

Human papillomaviruses (HPVs) are small DNA viruses that replicate in squamous epithelia. Specific types of HPV (high-risk HPVs) are the causative agents for cervical and several other cancers [1]. HPV infection normally initiates in the basal layer of the squamous epithelia and HPV genome is maintained in the basal cells. Extensive HPV genome amplification occurs in the mid and upper spinous strata. Under normal physiological conditions, squamous epithelia exit from cell cycle and undergo terminal differentiation. HPV infection alters epithelia differentiation. For example, epithelial cells infected by HPV type 16 (HPV-16), which is the cause of approximately 50% of cervical cancers worldwide, retain the ability to stratify but lose their ability to differentiate morphologically [2]. HPV also promotes S-phase re-entry in differentiating keratinocytes [3]. Hence, HPV can manipulate the cell cycle by establishing a milieu in the differentiated keratinocytes supportive for viral DNA amplification.

Although high-risk HPVs efficiently immortalize cultured primary human epithelial cells, they are not sufficient to induce transformation of human cells [4]. It is believed that abrogation of cell cycle checkpoint that leads to genomic instability by HPV in the basal epithelia enables cells to accumulate additional genomic aberrations necessary for malignant conversion [4]. The HPV E7 gene encodes a small protein of about 100 amino acids and together with E6, is consistently expressed in HPV-positive cervical cancers [5]. E7 degrades pRb and releases E2F to turn on genes required for DNA replication (reviewed in [6]). The expression of HPV E7 decouples the processes of cellular proliferation and differentiation [7]. HPV E7 plays an important role during the viral life cycle and the E7s encoded by high-risk HPV types contribute to the development of cancers.

Cell cycle progression is regulated by cyclins, cyclin-dependent kinases (Cdks) and their regulatory proteins at several checkpoints [8]. It is generally believed that serum-starved cells are in a G0 phase, a quiescent state of arrest for mammalian cells, where cells are proposed to be “out of the cycle” [9]. In quiescent state cells, the cyclin/Cdk inhibitor p27 is induced [10]. p27 was first identified as an inhibitor of cyclin E and Cdk2 complex and was found to act as a tumor suppressor [11]. Phosphorylation of p27 occurs at multiple sites and plays diverse roles in modulating its activity [10]. For example, phosphorylation of p27 at serine 10 (S10P) regulates its stability [12] or nuclear export [13], phosphorylation at threonine 198 (T198P) promotes cell migration [14] and interferes with p27's association with Cdk2 [15], phosphorylation at tyrosine 88 (Y88) prevents its inhibition on Cdk2 [16], phosphorylation at threonine (T187) affects its stability [17].

HPV E7 promotes cell proliferation under serum starvation conditions [18, 19] or in the presence of elevated levels of p27 [20], consistent with what was observed in cervical lesions [21]. How E7 expressing cells bypass p27-mediated cell cycle arrest has been a long-standing puzzle. An early study attributed p27 inactivation to direct physical interaction with E7 [22]. However, the fact that p27 is an abundant protein while the cellular level of E7 is relatively low makes this mechanism problematic. In other studies, it was proposed that cytoplasmic localization inactivates p27 [20, 23], but how E7 modulates p27 localization is not known. While phosphorylation of p27 at T157 (T157P) by Akt in E7 expressing cells has been detected and proposed as a mechanism for p27 localization in the cytoplasm [20, 23], the contribution of T157P to p27 localization and activities remains to be shown [23]. Furthermore, the extent of p27 cytoplasmic localization in HPV-infected cells remains to be established. Phosphorylation of p27 at other amino acid residues in HPV E7 expressing cells has not been reported.

Mirk/Dyrk1B is a dual function kinase with the ability to auto-phosphorylate on tyrosine and then phosphorylate other substrates on serine and threonine. Dyrk1B expression is low in most tissues and is elevated in many tumors [24, 25]. Dyrk1B was thought to play a role in maintaining the G0 quiescent state by phosphorylating and stabilizing p27 and destabilizing cyclin D1 [26, 27]. It is believed that arresting in a quiescent state allows cancer cells to survive suboptimal growth conditions [28]. Interestingly, knocking down Dyrk1B induced apoptosis and increased sensitivity of human cancer cells to therapeutic agents [29-32].

In the present study, we investigated the mechanism by which HPV-16 E7 abrogates the G0/G1 cell cycle checkpoint under serum starvation conditions. We showed that in both primary human keratinocytes (PHKs) and immortalized human epithelial cells, Dyrk1B was significantly up-regulated by high-risk HPV E7; moreover, depletion of Dyrk1B in E7-expressing cells impaired cell proliferation and caused cell cycle arrest under serum starvation conditions. Furthermore, Dyrk1B contributed to p27 phosphorylation at S10 as well as T198, which were important for entry into S-phase of E7 expressing cells. Moreover, we provide evidence that Dyrk1B upregulated HPV-16 E7 expression.

## RESULTS

### HPV E7 facilitates S phase entry with elevated cell cycle inhibitor p27

**Figure 1:**
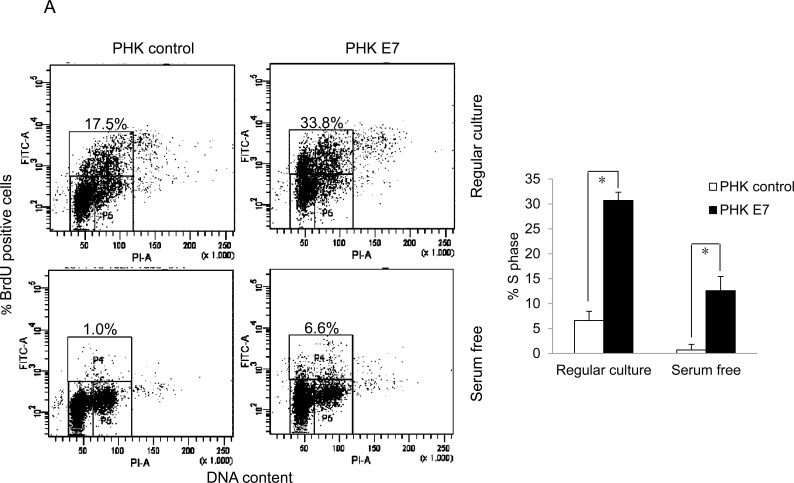

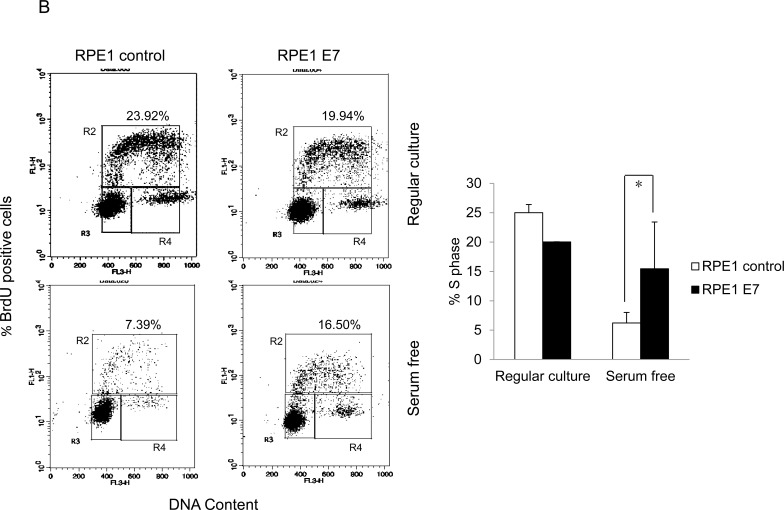

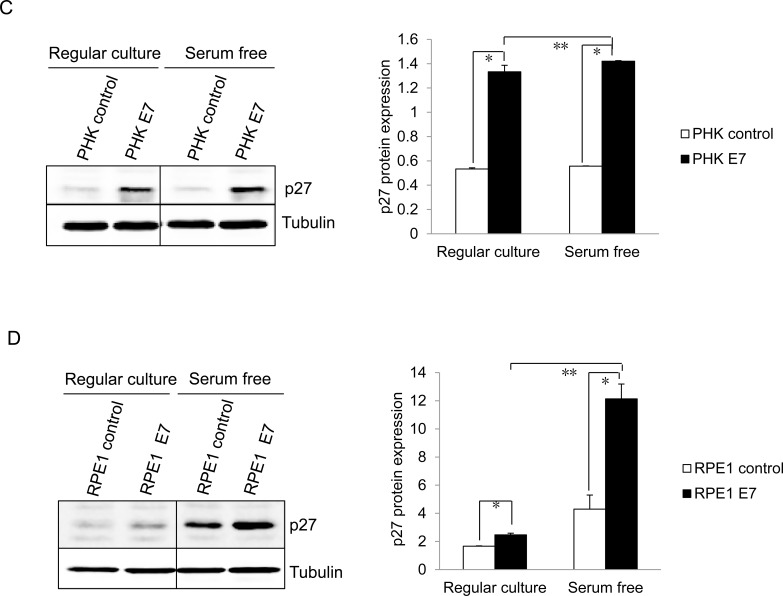

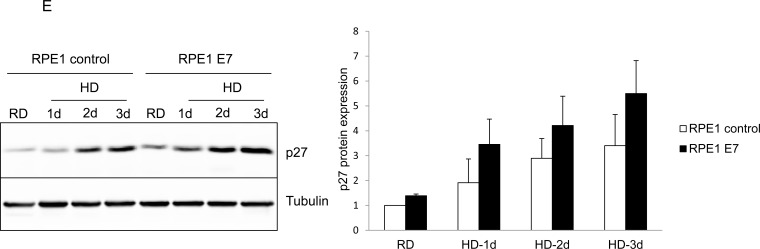
HPV E7 expressing cells entry into S phase under serum starvation and elevated levels of p27 Cells expressing E7 and control were cultured in the presence or absence of serum for 2 days. The cells were labeled with BrdU for 2 h before collecting and stained with 7-AAD and anti-BrdU antibody, analyzed by flow cytometry. BrdU-positive cells are gated and their percentages are indicated. **A.** PHKs. **B.** RPE1 cells. Lower panels, quantification of BrdU staining. Error bars reflect the standard deviations of the mean. **C.** and **D.** The steady-state levels of p27 in PHKs **C.** and RPE1 cells **D.** expressing E7 or control were examined by Western blot. **E.** RPE1 cells were plated at high density and the steady-state levels of p27 were examined. RD, regular cell density; HD, high cell density. β-tubulin was used as a loading control. A representative experiment of three is shown. Right panels, quantification p27 protein levels. *, *p* < 0.05; **, *p* < 0.01.

To examine the ability of HPV E7 to promote the proliferation of cells from a quiescent state, we measured BrdU incorporation of PHKs containing HPV-16 E7 (PHK E7) and control PHKs. Under regular culture conditions where the medium contains serum PHK E7 incorporated significantly more BrdU than control PHKs (Figure 1A). In the absence of serum, PHKs essentially stopped proliferating (Figure 1A). In contrast, PHK E7 continued incorporating BrdU, though the amount of BrdU incorporation was significantly reduced. These results are consistent with what was observed previously [18]. As proliferation and transfection efficiencies in PHKs are not satisfactory, we also employed the human retinal pigment epithelial RPE1 cells [33]. RPE1 cells expressing the wild-type E7 (RPE1-E7) and vector control (RPE1-vector) have been used in our recent HPV-related functional studies [34-36]. Similar to what was observed in PHKs, E7 expressing RPE1 cells expressed reduced steady-state levels of the tumor suppressor pRb [35] and incorporated significantly more BrdU than the vector control cells under serum starvation condition (Figure 1B). However, under regular culture condition there was no significant difference in BrdU incorporation between RPE1 cells expressing E7 and containing a vector.

We then examined the expression of p27, the major negative regulator of cell proliferation at quiescent state. p27 was previously known to be induced by serum starvation. The steady-state levels of p27 in both E7 expressing RPE1 and PHK cells are significantly higher than that of control cells (Figure 1C and 1D). The levels of p27 were further increased upon serum starvation in E7 expressing RPE1 cells (Figure 1D). In E7 expressing PHKs, although the level of increase in p27 upon serum starvation was limited, it was statistically significant (Figure 1C). Thus, we have demonstrated the ability of HPV E7 expressing cells to proliferate in the presence of elevated steady-state levels of p27 under serum starvation conditions. A previous study showed that HPV E7 expressing mouse fibroblasts proliferated at high density where elevated p27 was detected [20]. High cell density deactivate the mammalian target of rapamycin (mTOR) pathway to suppress the senescence program [37]. In RPE1 cells, p27 was also induced at high density in both vector control and E7 expressing cells, with the latter express more p27 (Figure 1E). The detailed mechanism by which E7 induces S-phase entry in the presence of elevated p27 is the subject of this study.

### Dyrk1B is up-regulated in HPV E7 expressing cells

**Figure 2:**
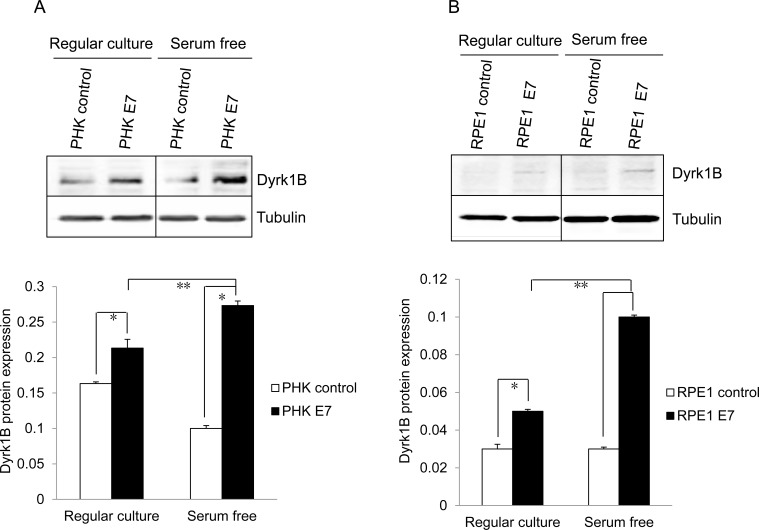

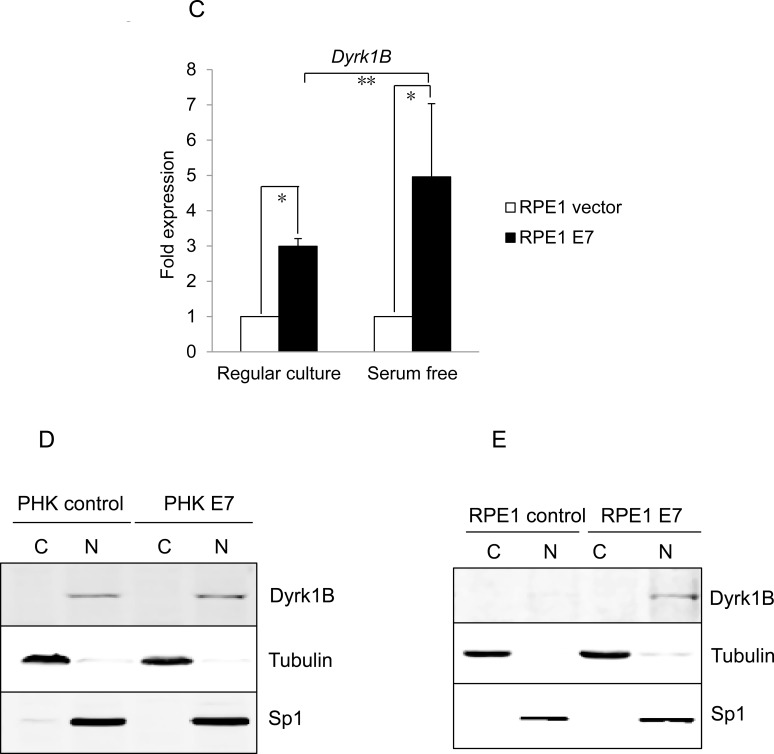
Dyrk1B expression and localization in HPV E7 expressing cells The steady-state levels of Dyrk1B in PHKs **A.** and RPE1 cells **B.** expressing E7 or control were examined by Western blot. β-tubulin was used as a loading control. Lower panels, quantification of Dyrk1B protein levels. **C.** *Dyrk1B* mRNA levels in RPE1 cells expressing E7 or control were examined by real-time PCR. Expression levels were assessed in triplicate and normalized to *GAPDH* levels. Results from three independent experiments were summarized. Cytoplasmic and nuclear fractions were prepared from PHKs **D.** and RPE1 cells **E.** expressing HPV E7 or control and immune-blotted with antibodies specific for Dyrk1B, β-tubulin (cytoplasmic protein marker) or SP1 (nuclear marker). Equal amount of cytoplasmic proteins and nuclear proteins were loaded. C: cytoplasm; N: nucleus. Data from one representative experiment of four are shown. *, *p* < 0.05; **, *p* < 0.01.

As an initial step toward understanding the mechanism by which E7 induces S-phase entry in quiescent cells, we examined the expression of Dyrk1B, the major kinase responsible for maintaining cells in the quiescent state. As shown in Figure 2A, the steady-state level of Dyrk1B was modestly but statistically significantly increased in E7 expressing PHKs as compared with control PHKs. Up-regulation of Dyrk1B protein also occurred in RPE1-E7 cells as compared with the vector control cells (Figure 2B). Upon serum starvation, while the steady-state levels of Dyrk1B did not change in the control PHKs or RPE1 cells, it was significantly increased in E7 expressing cells. Consequently, there was nearly 3-fold more Dyrk1B in E7 expressing cells compared with control cells. Consistently, mRNA for *Dyrk1B* was also increased in E7 expressing RPE1 cells compared with control cells (Figure 2C). Upon serum starvation, there was a further increase of *Dyrk1B* mRNA in E7 expressing RPE1 cells but not control cells (Figure 2C). These results are surprising, as Dyrk1B was reported to play a negative role in S-phase entry from quiescent state, while we have observed more E7 expressing cells incorporating BrdU with increased Dyrk1B expression (Figure 1). These data suggest that Dyrk1B may play a positive role in G0 to G1/S transition in E7 expressing cells. Notably, up-regulation of Dyrk1B in E7 cells is consistent with elevated levels of its phosphorylation substrate p27 (Figure 1C).

We then examined Dyrk1B cellular localization in E7 expressing cells to determine whether it is altered compared to control cells. Dyrk1B has been detected in both nucleus and cytoplasm in previous studies [31, 38-41]. We performed Western blot analysis following sub-cellular fractionation to determine and quantify the intracellular localization of Dyrk1B in E7 expressing and control RPE1 cells. Accordingly, nuclear and cytoplasmic proteins were prepared and analyzed. Successful fractionation was demonstrated by the expected sub-cellular localization of nuclear (SP1) and cytoplasmic (β-tubulin) protein markers (Figure 2D and 2E). Under our experimental conditions, the majority of Dyrk1B proteins were localized in the nucleus in both vector and E7 expressing cells. Therefore, there is no significant change in cellular localization of Dyrk1B in E7 expressing cells.

### Dyrk1B induces S-phase entry of quiescent E7-expressing cells

**Figure 3:**
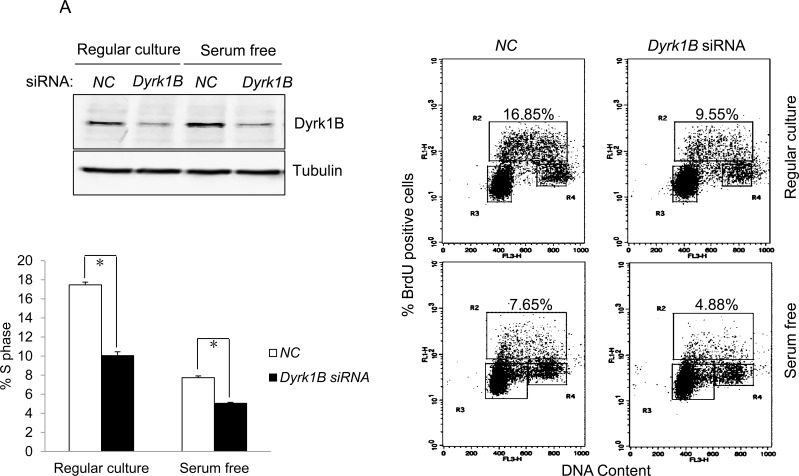

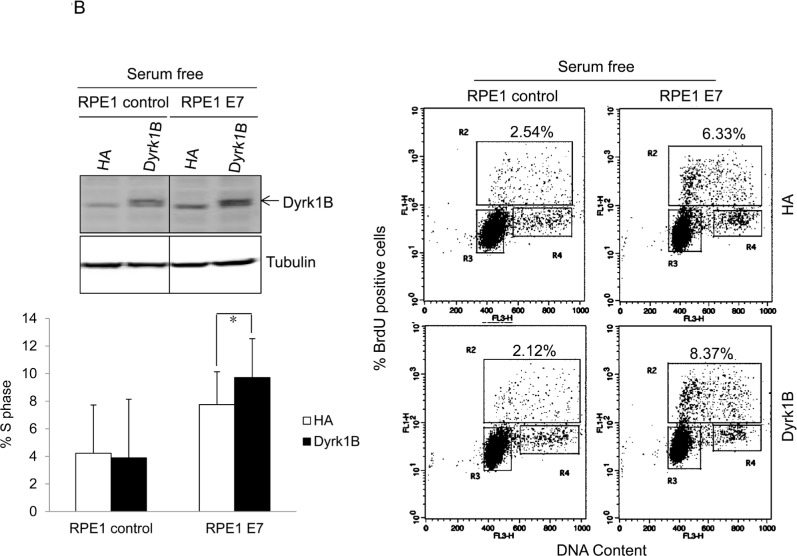

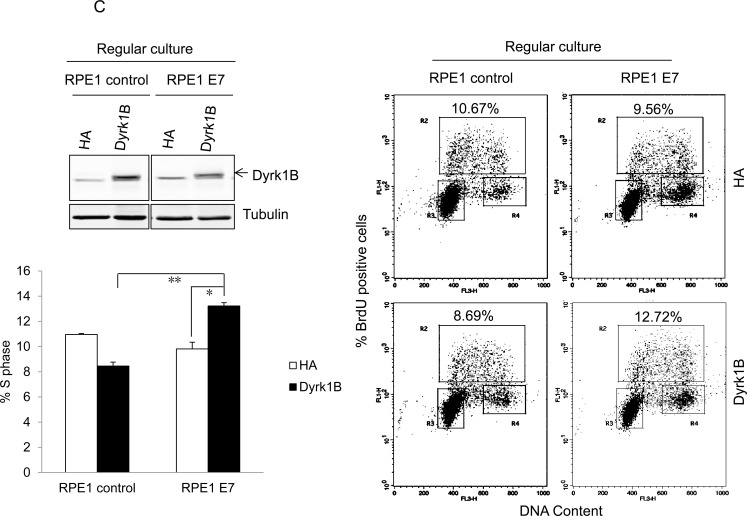
Dyrk1B promotes S phase entry in E7 expressing quiescent cells **A.** RPE1-E7 cells were transfected with siRNA targeting Dyrk1B and cultured in either regular medium or serum free medium. Forty-eight hours after transfection, cells in one set of dishes were harvested, lysed and Dyrk1B protein levels were analyzed by immunoblot. β-tubulin was used as a loading control. Cells in another set of dishes were labeled with BrdU for 2 hours before collecting. Cells were then stained with anti-BrdU antibody, counterstained with 7-AAD, and analyzed by flow cytometry and quantified (Lower left panel). The percentage of BrdU-positive cells was indicated. NC, non-silencing siRNA. **B.** RPE1-E7 and control cells were transfected with plasmids encoding HA-Dyrk1B fusion or control vector under either serum starvation **B.** or regular culture condition **C.**, and either were subjected to immunoblot analysis with anti-HA antibody, or labeled with BrdU and analyzed by flow cytometry and quantified (Lower left panel). Data from a representative of at least three experiments are shown.

To demonstrate the role of Dyrk1B in E7 induced S-phase entry from otherwise quiescent state, we used a siRNA targeting DYRK1B. The siRNA was previously demonstrated to down-regulate DYRK1B expression after transfection into cultured cells [38, 39]. Transfection of siRNA targeting DYRK1B reduced the steady-state levels of Dyrk1B in RPE1-E7 cells both in the presence or absence of serum (Figure 3A). We then used this siRNA to assess the role of Dyrk1B in E7-induced S-phase entry. Significantly, knocking down of Dyrk1B by siRNA led to a modest but statistically significant reduction of BrdU incorporation in RPE1-E7 cells, both under normal culture conditions and upon serum starvation (Figure 3A). These results suggested a positive role for Dyrk1B E7 in S-phase entry of E7 expressing cells from quiescent state.

To demonstrate the role of Dyrk1B in promoting S-phase entry of quiescent cells more directly, we transfected a plasmid encoding DYRK1B into E7 expressing and control RPE1 cells, and measured BrdU incorporation under serum starvation conditions. As shown in Figure 3B, ectopic expression of DYRK1B increased the number of E7 expressing RPE1 cells incorporating BrdU. Notably, the S-phase promoting effect of Dyrk1B was not limited to cells in quiescent state, as DYRK1B ectopic expression also increased BrdU incorporation of E7 expressing cells under regular culture condition (Figure 3C). These results demonstrate that in contrast to what was observed in other cell types, DYRK1B plays a positive role in G0/G1 transition in E7 expressing cells. Interestingly, DYRK1B overexpression did not increase S-phase entry in control RPE1 cells.

### Dyrk1B is important for p27 expression and phosphorylation

**Figure 4:**
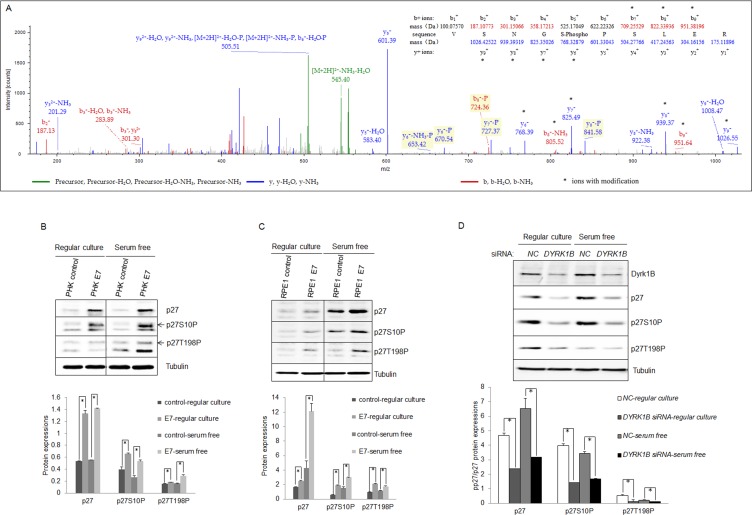
Dyrk1B was important for phosphorylation of p27 in HPV E7 expressing cells **A.** MS/MS spectra for peptide from human p27 spanning amino acid residues VSNGpSPSLER (6-15) in HPV-16 E7 expressing epithelial cells. Inset, amino acid sequence for the peptide. Masses that show an increase of amu are marked with an asterisk. **B.** and **C.** Expression and phosphorylation of p27 in HPV-16 E7 expressing cells. p27 and phospho-p27 levels in PHK **B.** and RPE1 **C.** cells expressing E7 or control were examined by Western blot and quantified (Lower panels). **C.** Dyrk1B is important for p27 phosphorylation in E7 expressing cells. RPE1-E7 cells were transfected with non-specific control (NC) or Dyrk1B specific siRNAs at a final concentration of 20 nM. One set dish of cells was cultured in serum-free medium. Forty-eight hours post-transfection, cells were harvested, lysed and protein levels were analyzed by immunoblot and quantified (Lower panel). β-tubulin was used as a loading control. A representative experiment of four is shown.

Since Dyrk1B is a kinase known to phosphorylate p27 at Serine 10, which could potentially inactivate p27 by causing its cytoplasmic localization [13], we examined p27 phosphorylation status in HPV E7 expressing cells. First we searched the raw data of mass spectrometry (MS) analysis of tryptic-digested peptides in the HPV-16 E7 expressing RPE1 cells. A phosphor-p27 peptide corresponding to amino acid residues VSNGpSPSLER (6-15) with a high score was identified. The mass spectrum of the peptide is shown in Figure 4A. The results suggest that serine 10 of p27 is phosphorylated in E7 expressing cells.

Next we examined p27 phosphorylation in E7 expressing cells by Western blot using a phospho-specific antibody to S10P. As shown in Figure 4B and 4C, p27 S10P are more abundant in E7 expressing cells than control cells (∼2-fold in RPE1 cells, ∼3-fold in PHKs). We also examined p27 phosphorylation at other known positions. Interestingly, p27 phosphorylation at T198 was also increased in E7 expressing cells (∼2.5-fold in RPE1 cells; ∼6-fold in PHKs). Phosphatase treatment essentially abolished the reactivity of the phosphospecific antibodies (not shown), indicating the specificity of the antibodies. However, in contrast to the previous observation [23], no significant difference in T157P between E7 and control cells was observed (not shown). In addition, no phosphorylation of p27 at Y88 or T187 in E7 expressing or control cells could be detected.

To verify that Dyrk1B is responsible for p27 S10P in E7 expressing cells, we knocked it down by siRNA. This reduction of *Dyrk1B* significantly reduced S10P in E7 expressing cells (Figure 4C). The steady-state levels of total p27 after *Dyrk1B* knockdown were also reduced. Since it was known that S10P contributes to the stability of p27, these results suggest that Dyrk1B is responsible for p27 S10P and p27 stability, though we cannot completely rule out the possibility that reduced p27 S10P is a result of reduced total amount of p27. Although direct phosphorylation of p27 by Dyrk1B has been demonstrated [26, 27, 42], we did not provid evidence that this occurs in E7 expressing cells. Notably, down-regulation of *Dyrk1B* also reduced the level of T198P, probably a result of S10P reduction, as it is required for T198P [43].

### Phosphorylation of p27 contributes to S-phase entry of quiescent E7 expressing cells

**Figure 5:**
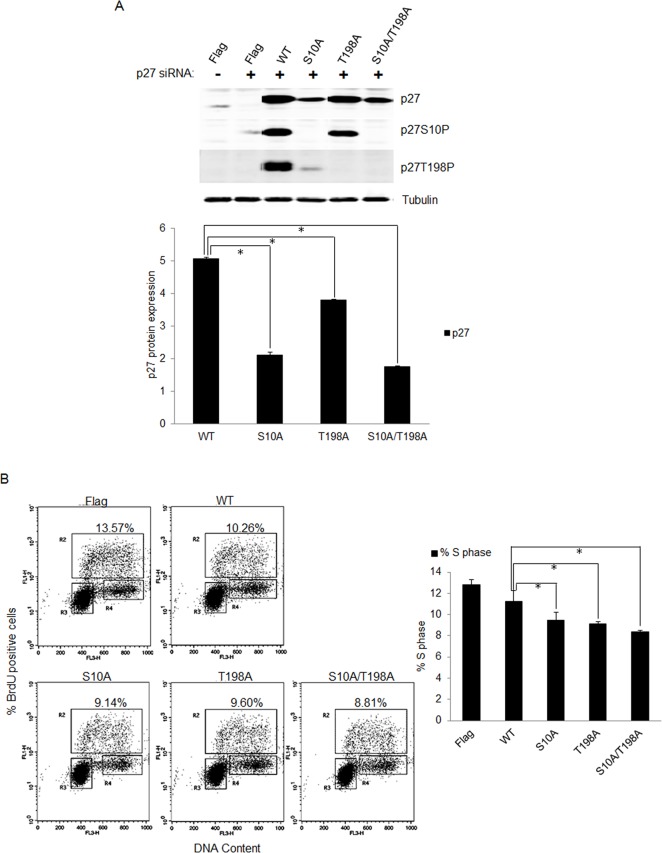
Phosphorylation of p27 was important for S-phase entry in E7 expressing quiescent cells **A.** RPE1-E7 cells were transfected with siRNA targeting p27 and plasmids encoding FLAG-tagged wild-type and mutant p27. Forty-eight hours post-transfection, cells were harvested, lysed and protein levels were analyzed by immunoblot and quantified (Lower panel). β-tubulin was used as a loading control. Cropped gels from a representative experiment are displayed. **B.** The above treated cells were stained with anti-BrdU antibody, counterstained with 7-AAD, and analyzed by flow cytometry and quantified (Right panel). The percentage of BrdU-positive cells was indicated. A representative experiment of three is shown.

To determine the role of p27 phosphorylation in E7 expressing cells, we constructed substitution mutations of p27 at S10 or T198 with alanine (S10A) and (T198A) to render them nonphosphorylatable. To facilitate detection, mutants were expressed as FLAG-tagged fusion proteins. The mutant p27, along with wild-type control, was then transfected into RPE1 cells expressing E7. Endogenous p27 were knocked down by siRNA. The transfected p27 were engineered to be siRNA resistant by change of nucleotides in the target sequence. In mutant transfected cells, p27 expression was examined by antibody against FLAG. Consistent with the notion that S10 phosphorylation contributes to p27 stability, the S10A p27 mutant was expressed to a much lower level as compared with wild-type p27 (Figure 5A). In T198A mutant cells, the level of T198A p27 was modestly reduced compared to wild-type p27, a result consistent with previous reports [40, 41]. As expected, the steady-state level of double mutant S10A/T198A in transfected cells was also significantly reduced. Notably, the antibody against p27 S10P did not detect any S10A mutant p27, and likewise, the antibody against p27 T198P did not detect any T198A mutant p27, indicating that these antibodies are indeed specific to phosphorylated p27 proteins.

The ability of the p27 mutant to arrest cells at the G0 checkpoint was then assessed following serum starvation and BrdU staining of transfected E7 expressing RPE1 cells. Transfection of cells with wild-type p27 reduced BrdU incorporation as expected (Figure 5B), while transfection of mutant p27 S10A resulted in significantly less BrdU incorporation compared with the wild-type p27. Similarly, transfection of T198A and double mutant S10A/T198A also reduced BrdU incorporation compared to wildtype p27 transfection. Notably, the effect of p27 mutants on BrdU incorporation was achieved with reduced level of p27 expression, which could underestimate their impact. These results suggest that phosphorylation of p27 at S10 and T198 are important for S-phase entry in E7 expressing cells.

### Dyrk1B up-regulates E7 expression

**Figure 6:**
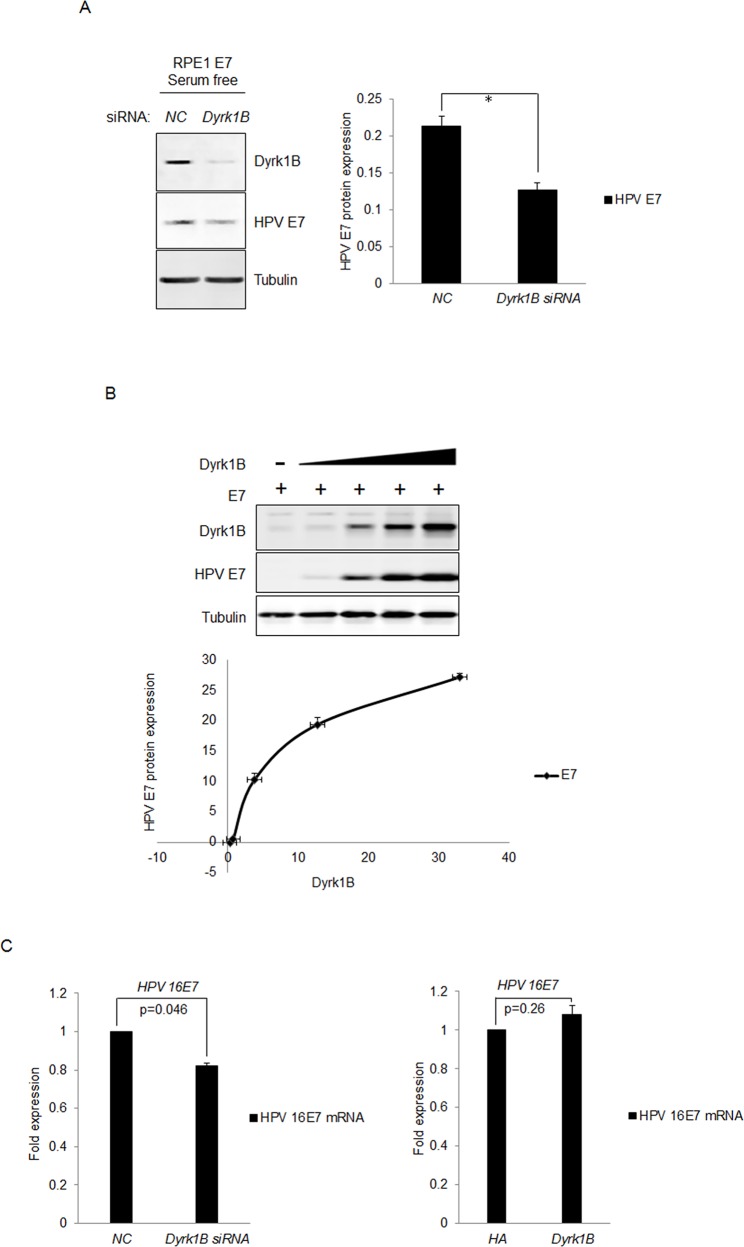
Dyrk1B increased the steady-state levels of HPV-16 E7 **A.** RPE1-E7 cells were transfected with siRNA targeting Dyrk1B and cultured in serum free medium and Dyrk1B protein levels were analyzed by immunoblot and quantified (Right panel). β-tubulin was used as a loading control. **B.** RPE1 cells were transfected with plasmids encoding HPV-16 E7 and varying amounts of plasmids encoding Dyrk1B. The steady-state levels of HPV-16 E7 as well as Dyrk1B were examined by Western blot and quantified (Lower panel). β-tubulin was used as a loading control. A representative experiment of four is presented. **C.** RPE1-E7 cells were transfected with siRNA targeting Dyrk1B (left panel) or the plasmid encoding HA-Dyrk1B fusion and mRNA were analyzed by Real-time PCR. The graphs represent data from an average of 3 independent experiments, each done in triplicate and normalized to *GAPDH* levels.

It was shown that the cellular homolog of Dyrk1B, Dyrk1A, interacted with and phosphorylated HPV-16 E7, which interfered with proteasome-dependent degradation of E7, and thus led to increased half-life and steady-state levels of E7 [44]. We therefore tested the possibility of Dyrk1B to increase the steady-state levels of E7 protein. For this, we transfected the siRNA targeting DYRK1B into RPE1-E7 cells and examined the steady-state levels of HPV-16 E7 by Western blot. Interestingly, as shown in Figure 6A, knocking down of DYRK1B significantly reduced the steady-state levels of E7. The results suggest that, similar to Dyrk1A, Dyrk1B also increases the steady-state level of HPV E7, which may contribute to the ability of Dyrk1B to promote S-phase entry of quiescent cells.

To directly demonstrate the ability of Dyrk1B to increase the steady-state levels of E7, we co-transfected plasmids encoding DYRK1B and HPV-16 E7 into RPE1 cells and determined the expression of E7 and DYRK1B. Transfection of DYRK1B modestly increased the protein levels of Dyrk1B in a dose-dependent manner (Figure 6B). Significantly, as levels of Dyrk1B increased, the steady-state levels of E7 also went up in a dose-dependent manner. Taken together, these results demonstrated that Dyrk1B up-regulates E7. The upregulation of E7 is probably not due to a control at the transcriptional level, as the drop of E7 mRNA level was minimal after knocking down *Dyrk1B* (Figure 6C, left panel). Consistent with these results, transfection of *Dyrk1B* did not significantly increase E7 mRNA level (Figure 6C, right panel).

## DISCUSSION

In this study, we investigated the mechanism by which HPV-16 E7 abrogates the G0/G1-S cell cycle checkpoint. We showed that the dual-specificity kinase Dyrk1B was upregulated in the high-risk HPV-16 E7 expressing cells and played an important role in abrogation of the G0/G1/S checkpoint. We further showed that Dyrk1B played a role for elevated phosphorylation of Cdk inhibitor p27, which contributed to its inactivation in E7 expressing cells. Dyrk1B also upregulated the expression of E7. Taken together, this study uncovered a novel function of Dyrk1B in high-risk HPV E7-mediated cell proliferation and established the role of p27 phosphorylation in promoting cell proliferation in E7 expressing cells. This study helps resolve a long-standing mystery regarding how cells expressing HPV E7 proliferate in the presence of abundant Cdk inhibitor p27.

It was known that HPV E7 promotes cell proliferation in the presence of elevated levels of p27 [20]. This activity of E7 is probably shared between both low-risk and high-risk HPVs. However, the high-risk HPV E7 may possess stronger ability to promote cell proliferation that may contribute to carcinogenesis originated in the basal epithelial. Results from this study demonstrated that S10P and T198P contributed to inactivation of p27; Dyrk1B contributed to p27 phosphorylations and S-phase entry. These observations provide mechanistic insight into E7 inactivation of p27.

Dyrk1B was thought to play a role in maintaining the G0 quiescent state by phosphorylating and stabilizing p27 as well as destabilization of cyclin D1 [26, 27, 42]. In contrast, we provide evidence that Dyrk1B plays a role in promoting S-phase entry from quiescent state. This observation is consistent with the observation that Dyrk1B expression is low in most tissues and is elevated in several solid tumors [25]. The discrepancy between the previous studies and our current observation regarding the function of Dyrk1B might be explained by differences in experimental conditions. Alternatively, the discrepancy could simply be due to differences in data interpretation. In one early study, colon carcinoma cells transfected with *Dyrk1B* exhibited increases in cell number compared with controls under serum starvation condition [45]. However, such a pro-cell proliferation activity of Dyrk1B has been interpreted as a pro-survival function [24]. There is a possibility that HPV E7 inactivates pRb, reduces the requirement for the Dyrk1B target cyclin D1, and thereby reveals positive impact of Dyrk1B on cell cycle that would have been masked by its negative effect. In addition, Dyrk1B up-regulates E7 expression, which is unique to other cell types that do not express E7. These findings have important clinical implications, as Dyrk1B may serve as a target for therapy in HPV-associated cancers. Dyrk1B has not been studied in the context of HPV. How Dyrk1B is upregulated by E7 remains to be studied. The expression of Dyrk1B in the cervix, including cervical cancer, awaits exploration.

It was shown that phosphorylation of p27 at S10 regulates its stability [12] and nuclear export [13], and that phosphorylation at T198 interferes with its association with Cdk2 [15] and promotes its cytoplasmic localization [46]. On the other hand, Cdk2 was previously thought to be essential for the G1/S transition while Cdk1 functions only at the G2/M progression [47]. Subsequent studies have shown a role for Cdk1 at the G1 checkpoint in the absence of Cdk2 in mice [48]. Our recent studies have demonstrated a role for Cdk1 at the G1 checkpoint in the presence of Cdk2 in human cells [49]. The role for Cdk1 at G0/G1/S transition remains to be established. Future studies will determine the Cdk that is important for S-phase entry from quiescent state in E7 expressing cells.

p27 was originally proposed to be a tumor suppressor. Its oncogenic function has not been explored thoroughly, especially in HPV oncogene expressing cells. Tumorigenic properties of p27, such as enhancing cell mobility when localized in the cytoplasm, have also been demonstrated [50-52]. In human cancers, a correlation of cytoplasmic localization of p27 with high tumor grade and poor prognosis was discovered [53]. Cytoplasmic localization of p27 has also been observed in cervical and oral cancers [20, 23, 54]. Future studies should explore the function of p27 when localized in the cytoplasm in cell migration/invasion.

While this study focused on functional interactions of HPV E7 with Dyrk1B and p27, we should also pay attention to its cellular homolog Dyrk1A. Interestingly, Dyrk1A phosphorylated HPV-16 E7 and increased half-life and steady-state levels of E7 [44]. On the other hand, E7 functionally antagonized the activity of Dyrk1A, which phosphorylates LIN52 to assemble the DREAM complex [55]. DREAM binds CDE and CHR promoter elements [56]. HPV E7 perturbs DREAM, affects its DNA binding, leading to deregulation of cell cycle genes and ultimately the cell cycle [57, 58, 59]. Of particular interest, Poly-like kinase 4 (PLK4) is among the genes regulated by DREAM and upregulated by E7. The HPV-16 E7 oncoprotein induces centriole multiplication through deregulation of PLK4 expression [60].

In summary, we demonstrated that Dyrk1B was upregulated by high-risk HPV E7. Dyrk1B was important for G0/G1/S cell cycle progression in HPV E7-expressing cells. Dyrk1B contributed to p27 phosphorylation in E7 expressing cells and upregulated E7. These results suggest a role for Dyrk1B in HPV E7 expressing cells that may contribute to HPV-induced carcinogenesis. Dyrk1B may serve as a target for therapy in HPV-associated cancers.

## MATERIALS AND METHODS

### Cell culture

PHKs were derived from neonatal human foreskin epithelium obtained from the University of Massachusetts Hospital as described [61]. PHKs were maintained on mitomycin C-treated J2-3T3 feeder cells in F-medium composed of 3 parts Ham's F12 medium (Invitrogen), 1 part DMEM (Invitrogen), and 5% fetal bovine serum (Invitrogen) with all supplements described [62]. RPE1 cells [33] were maintained in a 1:1 blend of DMEM and Ham's Nutrient Mixture F12 medium (Invitrogen) plus 10% FBS. All cells were grown in a 5% CO_2_ atmosphere at 37°C in above medium with the addition of 100 units/ml of penicillin and 100 μg/ml streptomycin.

PHKs and RPE1 cells expressing HPV-16 E7 or vector were established by retrovirus-mediated infection using the pBabe-puro-based retroviral vector as described [36]. Experiments were performed with cells within 15 passages. For high cell density analysis, RPE1 cells were plated at 100,000 (Rugular density) and 2 million (High density) per 6 cm dish. Cells at regular density were collected when they became ∼60% confluence; for cells at high density, they were counted as day 1 once, they became confluent. Cell extracts were prepared in 1, 2 and 3 days.

### Western blot and cell fractionation

For Western blot analysis, protein were analyzed as described [36]. The following antibodies were used: Dyrk1B (Cell Signaling Technology), β-Tubulin (Sigma, T5168), p27 (Santa Cruz, sc-528), p27 phospho S10 (Abcam, ab62364), p27 phospho T198 (R&D Systems, AF3994), FLAG (Sigma, F1804), HA (Santa Cruz, sc-7392), HPV16 E7 (Santa Cruz, sc-6981), IRDye 800CW goat anti-mouse IgG (Licor, 926-32210), IRDye 800CW goat anti-rabbit IgG (Licor, 926-32211). The bound complex was detected using the Odyssey Infrared Imaging System (Li-Cor Lincoln, NE). The images were analyzed and quantified using the Odyssey Application Software, version 3.0 (Li-Cor). Intensity of bands was normalized in relative to tubulin signals.

Subcellular fractions were prepared using the ProteoExtract subcellular proteome extraction kit (Calbiochem) according to the manufacturer's instructions. Briefly, cytoplasmic, soluble, and insoluble nuclear extracts were prepared using the hypotonic buffer, hypertonic buffer, and insoluble buffer, respectively. Equal volume of cytoplasm and nuclear extracts was obtained. SP1 and β-tubulin were used as loading controls for the nuclear and cytoplasmic fractions, respectively.

### RNA extraction and RT-PCR analysis

RNA extraction was carried out using the TRLzol Reagen (Invitrogen) according to the manufacturer's instructions. cDNA was synthesized using random primers with the PrimeScript™ RT Reagent Kit with gDNA Eraser (Takara) according to manufacturer's instructions. Amplification of PCR products was quantified using SYBR® Premix Ex Taq™ (Takara) and monitored on a DNA Engine Peltier thermal cycler (Bio-Rad) equipped with a Chromo4 real-time PCR detection system (Bio-Rad). The following cycling conditions were used: initial denaturation at 95°C for 3 min, followed by 40 cycles of 95°C for 15 s, 59°C for 30 s, and 72°C for 30 s using primer sets for *Dyrk1B* (forward, 5′- ATTCACTGCGACCTCAAG-3′; reverse, 5′- GCGGCTCTGGATATACTG-3′), *HPV 16E7* (forward, 5′- AGTGTGACTCTACGCTTCGGTTG-3′; reverse, 5′-CTGAGAACAGATGGGGCACAC-3′), and *GAPDH* (forward, 5′-GCACCGTCAAGGCTGAGAAC-3′; reverse, 5′-TGGTGAAGACGCCAGTGGA-3′). Expression levels were assessed in triplicate and normalized to *GAPDH* levels, and graphs represent the combined results for three independent biological replicates.

### siRNA transfection

RPE1 cells were seeded in 6-cm dishes at 3 × 10^4^ cells and cultured in medium without antibiotics for 24 h. Chemical modified siRNA were purchased from Guangzhou RiboBio (RiboBio, Guangzhou, China) and transfected into cells using Lipofectamine 2000 (Invitrogen, Life Technologies, CA, USA) as recommended by the manufacturer. Cells were transfected with siRNA at a concentration of 20 nM. The sequences of siRNAs are as follows: si-p27: 5′-CCGACGAUUCUUCUACUCA-3′ (Purchased from Guangzhou RiboBio (Guangzhou, China); si-*Dyrk1B*: 5′-CGAAAGAACUCAGGAAGGA-3′, as described [30]. Cells were harvested and used for further experiments 48 h after transfection.

### Plasmids and transfection

The plasmid pCMV-3FLAG-16E7 encodes HPV-16 E7 with a FLAG tag at the N-terminus in vector pCMV-3Tag-1. The plasmid pHA-Dyrk1B encodes human Dyrk1B with the C-terminal HA epitope tag in vector pcDNA3. The plasmid pFLAG-p27-WT encodes human p27 with the FLAG epitope tag at the N-terminus. To construct p27 mutants and siRNA-resistant variants that contain sequence CCGACGATTCTTCTACTCA from nucleotide (nt) 1040 to 1058, mutagenesis was performed using the QuikChange site-directed mutagenesis kit and QuickChange XL site-directed mutagenesis kit (Stratagene). The plasmid pFLAG-p27-S10 encodes p27 with S10A change (AGC to GCC from nt 598 to 600) while pFLAG-p27-T198A encodes p27 T198A alteration (ACG to GCG from nt 1162 to 1164). Mutations were confirmed by DNA sequencing. Transient transfection was performed with Lipofectamine 2000 (Invitrogen, Life Technologies, CA, USA) using 2 μg of pHA-Dyrk1B.

### Flow cytometry

For BrdU labeling experiment, BrdU (final 20uM) was added to the medium for 2 h before cells were collected and cells were harvested and fixed in 70% ethanol. The cells were permeabilized with 2 N HCl–0.5% Triton X-100, neutralized with 0.1 M sodium tetraborate, stained with monoclonal anti-BrdU (BD Biosciences), and then with anti-mouse IgG F(ab)2-FITC (Sigma), and counterstained with PBS-7-AAD-RNase A. Measurements of the immunofluorescent cells were performed with a FACS Calibur analyzer (BD) and analyzed with FCSexpress software.

Flow cytometric analysis was performed on a BD FACSAria™ III sorter instrument equipped with BD FACSDiva™ 7.0 software (BD Biosciences, New Jersey, USA). FITC 490nm fluorescence was acquired in logarithmic amplification in FL1 and 7-AAD 650nm fluorescence was acquired in linear amplification in FL3.

### Mass spectrometry

Sample preparation and immunoprecipitation were performed as described [63]. The soluble proteins were separated by electrophoresis on 12% SDS-polyacrylamide gels. Proteins in gel were stained with Coomassie brilliant blue and then destained with 30% ethanol and 10% acetic acid. Selected bands corresponding to the known molecular weight of p27 were excised from the gel with a sterile blade and subjected to a standard in-gel digest protocol as described [64]. The digests were analyzed by LC-MS/MS using a Thermo EASY-nLC II system (Thermo Scientific, Hvidovre, Denmark) that was interfaced to a LTQ Orbitrap Elite/Velos Pro mass spectrometer (Thermo Scientific). LC MS/MS files exported from XCalibur were searched as described [63]. Protein identification was subjected to strict data QC filtering within MASCOT and SQUEST. In the case of p27 Ser10 containing-peptide VSNGSpPSLER (Amino acids 6-15), the Ser10 site was detected only in the phosphorylated form and full mass spectra (MS) were recorded on the peptides over a 100 to 2000 m/z range followed by MS/MS of m/z 1026.42522 [64].

### Statistical analysis

Data were presented as mean ± standard deviation. Data sets were graphed and analyzed by the two-tailed Student's *t* test. *p* ≤ 0.05 was considered statistically significant.
